# Cartilaginous conundrums: an integrated approach to synovial chondromatosis diagnosis

**DOI:** 10.11604/pamj.2025.52.128.43324

**Published:** 2025-11-26

**Authors:** Suhit Naseri, Kishor Hiwale

**Affiliations:** 1 Department of Pathology, Jawaharlal Nehru Medical College, Sawangi, India

**Keywords:** Cartilage diseases, integrated approach, diagnostic imaging

## Image in medicine

Synovial chondromatosis is a rare benign disorder characterized by the development of numerous cartilaginous nodules within the synovial membrane of joints, primarily affecting the knee. The typical onset is in the 30s-50s, with an incidence rate of 1 in 100,000 and a male-to-female ratio of 1.8: 1. We report a case of a 24-year-old male patient who presented with progressively worsening right knee pain, swelling, and limited range of motion persisting for over a month. There was no history of trauma or previous joint disorders. The skin on the surface of the mass was free from redness, swelling and ulceration, and no venous bulging. Initial radiographic imaging of the knee showed multiple intra-articular calcified bodies, suggestive of synovial chondromatosis. Magnetic resonance imaging (MRI) further characterized the extent of synovial involvement and the presence of loose bodies within the joint space. Synovial fluid analysis was unremarkable, ruling out infection or inflammatory arthritis. Arthroscopic removal of the swelling revealed synovial hypertrophy and multiple cartilaginous nodules ranging in size from a few millimeters to several centimeters. The nodules were firm and lobulated, with a smooth and glistening surface. Histopathological examination confirmed the diagnosis of synovial chondromatosis, demonstrating clusters of mature hyaline cartilage with focal areas of calcification. Synovial chondromatosis is a rare but important differential diagnosis in patients presenting with joint pain and swelling, particularly in the absence of trauma or inflammatory conditions. Early recognition, accurate diagnosis, and appropriate management, including surgical intervention when indicated, lead to favorable outcomes and improved quality of life for affected individuals.

**Figure 1:**
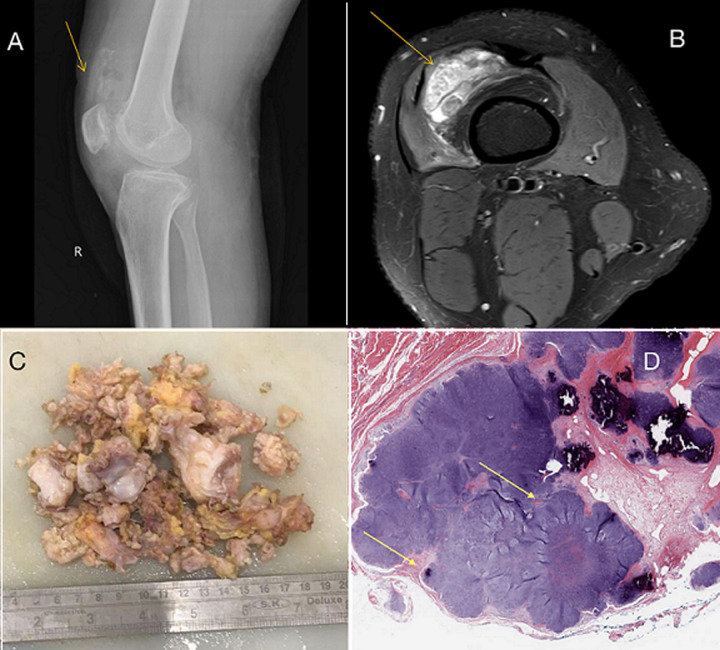
A) X-ray of right knee (lateral view) depicting multiple condromatosis; B) axial magnetic resonance imaging sections showing multiple loose bodies around the knee; C) gross image of the lesion showing surgically excised multiple synovial chondromatosis lesions; D) microscopy of the lesion showing multiple hyaline cartilage nodules

